# Left Ventricular Noncompaction—A Systematic Review of Risk Factors in the Pediatric Population

**DOI:** 10.3390/jcm10061232

**Published:** 2021-03-16

**Authors:** Katarzyna Łuczak-Woźniak, Bożena Werner

**Affiliations:** 1 Department of Pediatric Cardiology and General Pediatrics, Doctoral School, Medical University of Warsaw, 02-091 Warsaw, Poland; katarzyna.luczak-wozniak@wum.edu.pl; 2 Department of Pediatric Cardiology and General Pediatrics, Medical University of Warsaw, 02-091 Warsaw, Poland

**Keywords:** left ventricular noncompaction, hypertrabeculation, noncompaction cardiomyopathy, children, adolescents

## Abstract

Left ventricular noncompaction (LVNC) is a heterogeneous, often hereditary group of diseases, which may have diverse clinical manifestations. This article reviews the risk factors for unfavorable outcomes of LVNC in children, as well as discuss the diagnostic methods and the differences between pediatric and adult LVNC. Through a systematic review of the literature, a total of 1983 articles were outlined; 23 of them met the inclusion criteria. In echocardiography the following have been associated with adverse outcomes in children: Left ventricular ejection fraction, end-diastolic dimension, left ventricular posterior wall compaction, and decreased strains. T-wave abnormalities and increased spatial peak QRS-T angle in ECG, as well as arrhythmia, were observed in children at greater risk. Cardiac magnetic resonance is a valuable tool to identify those with systolic dysfunction and late gadolinium enhancement. Genetic testing appears to help identify children at risk, because mutations in particular genes have been associated with worse outcomes. ECG and imaging tests, such as echocardiography and magnetic resonance, help outline risk factors for unfavorable outcomes of LVNC in children and in identifying outpatients who require more attention. Refining the current diagnostic criteria is crucial to avoid inadequate restrain from physical activity.

## 1. Introduction

Left ventricular noncompaction (LVNC) is a heterogeneous group of diseases morphologically characterized by excessive trabeculations with concomitant deep recesses predominantly present in the left ventricle of the heart [1]. The significance of this finding in imaging studies has been widely debated mostly due to the polymorphic course of the disease, ranging from asymptomatic patients to children who die or undergo cardiac transplant during the first five years after diagnosis, which has been reported in around 6% of cases [2]. Furthermore, the placement of LVNC among cardiomyopathies is inconsistent; on the one hand, the American Heart Association (AHA) lists LVNC as a type of genetic cardiomyopathy together with hypertrophic and arrhythmogenic right ventricular cardiomyopathy [3]. On the other hand, the European Society of Cardiology (ESC) labels LVNC as an unclassified type of cardiomyopathy [4].

Some argue that the current definition of LVNC requires revision, because it is limited only to morphology and does not consider the function of the ventricle or the kind of genetic mutations; thus, suggestions regarding the division of LVNC into subtypes have been made [1,2,5,6]. Towbin et al. [1,5] outlined a classification of LVNC into the following: (1) Isolated LVNC (with normal cardiac function); (2) LVNC with congenital heart diseases (CHD); (3) HCM (hypertrophic cardiomyopathy) with LVNC; (4) DCM (dilated cardiomyopathy) with LVNC; (5) RCM (restrictive cardiomyopathy) with LVNC; (6) HCM-DCM with LVNC; (7) right ventricular noncompaction; (8) biventricular LVNC; and (9) LVNC with arrhythmia.

In children, the incidence of LVNC is estimated to be 0.11 per 100,000, with the highest incidence below one year of age, and children with isolated LVNC and normal ejection fraction are usually diagnosed at an older age [2,7]. With the improving imaging techniques and greater availability of echocardiography and cardiac magnetic resonance (CMR) the diagnosis is more frequent, and thus, the incidence will increase, with the ensuing risk of overdiagnosis and overtreatment [8]. For this reason, there is an urgent need to outline risk factors, which could help to successfully determine patients who require regular controls, and to concurrently delineate patients who can safely undertake physical activity.

This review presents current knowledge concerning the risk factors for an unfavorable outcome of LVNC in the pediatric population, and discusses the diagnostic methods and differences between pediatric and adult LVNC. To our knowledge, no systematic reviews concerning pediatric LVNC and the variety of its risk factors have been published to date.

## 2. Material and Methods

**Figure 1:**
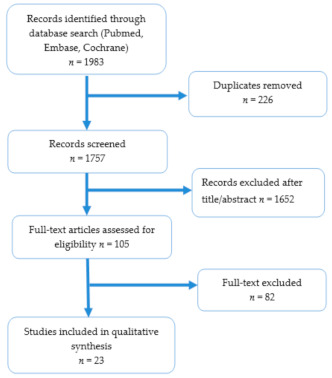
PRISMA search scheme; *n* = number of students.

A computer search was conducted according to the Preferred Reporting Items for Systematic Reviews and Meta-Analyses (PRISMA) scheme by two independent observers in three major databases (Pubmed, Embase, Cochrane) using the following search terms: (left ventricular noncompaction OR noncompaction OR hypertrabeculation OR LVNC OR NCLV) AND (children OR pediatric OR paediatric or neonate* OR infant* or adolescent*) AND (event* OR MACE OR major adverse cardiovascular event OR heart failure OR heart transplantation OR ICD OR ventricular arrhythmia OR ventricular tachycardia OR ventricular fibrillation OR survival OR outcome OR death OR mortality OR thromboembolism OR stroke) (Figure 1). Articles in which authors outlined risk factors for unfavorable outcomes in pediatric LVNC, such as the following: Progression of heart failure, malignant ventricular arrhythmia (sustained ventricular tachycardia (VT), ventricular fibrillation (VF), or appropriate ICD (implantable cardioverter defibrillator) shock), stroke, cardiac arrest, sudden death, implantation of LV assistance device, ICD implantation or heart transplant were used in our analysis. In our review, we included studies on children and adolescents (from birth to 21 years old). Publications in which either comparison between outcomes in two groups, Kaplan-Meier survival curves, univariate, or multivariate regression analysis was performed were taken into consideration.

Case reports, conference abstracts, meta-analyses, systematic reviews, and articles written in languages other than English were excluded from the analysis. To avoid data duplication, publications from the same centers on the same numbers of patients, or on similar numbers of patients with the same conclusions are presented in the summary tables (Tables 1 and 2) only once and were counted only once. One study was excluded, due to the inconsistency in the numerical data. Reports on both adult and pediatric populations, in which data were summed and where it was impossible to differentiate risk factors between the two groups, were not taken into consideration. Articles concerning genetic risk factors were not included in the analysis, due to the recently published meta-analysis by van Waning et al. [9]. The selected studies were independently analyzed by two researchers. The potential risk factors for unfavorable outcomes of LVNC (such as: Cardiac death, implantation of LV assistance device, heart transplant, sustained ventricular tachycardia, ICD implantation, or appropriate ICD shock) were divided into five major categories: Findings related to ECG (electrocardiography), echocardiography, CMR, coexisting heart diseases and other findings. Due to various inclusion criteria, data presentation, and statistical approaches in the outlined articles, no statistical meta-analysis was performed.

## 3. Results

Altogether 23 out of 1983 articles were considered (Figure 1); no additional records were found through other searches. There were 226 duplicates identified, 1652 records were excluded based on their titles or abstracts, and 82 were eliminated after a full-text revision. The articles included in this review were published between 2004 and 2020 and involved 1812 children with LVNC. The risk factors outlined in the publications are listed in Tables 1–3. Altogether 214 children died (11.8%), and 104 (6%) underwent a heart transplant, and the mean observation time in the outlined publications was 33 months, ranging from 0 to 322 months.

### 3.1. Echocardiography

Four different echocardiographic diagnostic criteria for LVNC have been used in the studies included in this review (Table 1 and Table 2). In four studies, it was not specified, which criteria were used. Moreover, in four studies, more than one diagnostic approach has been used.

In 14 studies of 1081 children, authors analyzed systolic dysfunction of the left ventricle as a risk factor; in 12 of the lower left ventricular ejection fraction (LVEF) or shortening fraction (FS) was associated with a worse outcome (Table 1 and Table 2). The lower systolic function of the left ventricle was a risk factor in both children with LVNC without congenital heart diseases (Shi et al. [7], Wang et al. [10], Brescia et al. [11]), as well as in studies including patients with other heart defects (Hirono et al. [12], Hirono et al. [13], Rodriguez-Fanjul et al. [14], Arunamata et al. [15], van Waning et al. [16], Zuckerman et al. [17], Ozgur et al. [18], Punn et al. [19], McMahon et al. [20]). In studies by Gan et al. [21], and Tsai et al. [22], however, LVEF did not prove to be a risk factor. It is of note, that various authors used different cut-off values for significance of left ventricle dysfunction ranging from LVEF < 24% to LVEF < 55%, as well as EF or FS, analyzed as continuous variables in some studies. In the study by Wang et al. [10], performed on over 200 pediatric patients with LVNC (without congenital heart diseases), reduced LVEF was correlated with decreased thickness of the compacted layer in the left ventricular posterior wall, and it was a predictor of death, transplantation, or ICD implantation.

Greater left ventricular end-diastolic dimension (LVEDD) was reported as an unfavorable risk factor in children in five studies of 263 children (Hirono et al. [13], Shi et al. [7], Arunamata et al. [15], Zuckerman et al. [17], and Wald et al. [23]), with various data presentation by different authors (i.e., cut off-values ranging from z-score > 2 to z-score > 8.56; comparison between adverse and benign groups, or hazard ratios with confidence intervals) (Table 1 and Table 2). In the study by MacMahon et al. [20], reduced early diastolic tissue Doppler velocity (e’) at the lateral mitral annulus was found to be an independent predictor of death or heart transplant. Rodriquez-Fanjul et al. [14], in their report on 14 neonates, 13 of whom had coexisting CHD, mentioned biventricular involvement to be a risk factor for death.

The role of the noncompaction-to-compaction (NC/C) ratio in echocardiography was addressed in seven studies, with a variety of different conclusions, cut-off values for statistical significance, as well as mean NC/C ratios given [7,10,13,17,19,21,23]. In the study by Hirono et al. [13] on 53 children with LVNC and concomitant congenital heart diseases an NC/C ratio >8.33 was a risk factor for death; correspondingly, Wald et al. [23] also suggested adverse outcomes (such as: death, heart transplant, or transplant listing) in patients with NC/C >3. In the study by Gan et al. [21] on 47 patients with isolated LVNC, worse survival was observed in patients with NC/C > 2. Punn et al. [19], in their study including children with CHD, described more segments involved in patients who underwent heart transplant or died; however, no relationship was found with the NC/C ratio. In the study by Shi et al. [7] on children without CHD, the NC/C ratio was borderline significant in terms of outcome (*p* = 0.07). No correlation between outcome and NC/C ratio was found in studies by Wang et al. [10] (who analyzed mean NC/C ratio) and Zuckerman et al. [17] (NC/C ≥ 2:1).

In a recent publication by Arunamata et al. [15], which included children with CHD, the significance of speckle tracking echocardiography was raised because radial, circumferential and longitudinal strain were significantly lower in children with LVNC and adverse outcomes, such as: heart transplant or death.

### 3.2. Cardiac Magnetic Resonance Imaging

Altogether in three studies, the role of CMR was addressed (Table 1 and Table 2). In the study by Cheng et al. [29] on adolescents without CHD (mean age 14 years), LGE (late gadolinium enhancement) was a predictor of adverse outcomes (such as death or heart transplant). Cortez et al. [26], in their study on younger children without congenital heart diseases (mean age 1 year) did not outline LGE as a risk factor for sustained ventricular tachycardia, however, they observed greater LVEDVi (indexed left ventricular end-diastolic volume) in patients with this type of arrhythmia. Van Waning et al. [16], in their analysis of both echocardiographic and CMR results, suggested an association between LV systolic dysfunction and worse outcome in children (including those with CHD); the role of LGE was not assessed.

### 3.3. Electrocardiography and Arrhythmia

Table 3 shows the ECG results that were presented in 12 studies [10,11,12,13,14,16,21,22,23,24,26,29]; abnormal ECG findings were present in 56–100% of children with LVNC, with the most common being the following: Abnormal T-wave, the fulfillment of the voltage criteria for ventricular hypertrophy, and ST-abnormalities. In the study by Brescia et al. [11] on 242 children without CHD T-wave inversion and ST-segment abnormalities were noted as a risk factor of death or transplantation. Hirono et al. [12] mentioned T-wave abnormalities in first graders to be associated with worse outcomes, such as: Death or heart transplant. Moreover, in the study by Cortez et al. [26] on children without CHD, higher heart rate and spatial QRS-T angle ≥147° in children with LVNC were said to be risk factors for sustained ventricular arrhythmia.

Howard et al. [24] showed a relationship between Wolf-Parkinson-White (WPW) pattern on ECG and lower LVEF in children with isolated LVNC; the latter improved in some children after catheter ablation. No statistical significance in terms of survival was observed between children with LVNC and WPW and those without WPW.

The occurrence of arrhythmias in children with LVNC varied from 5% to 34%, depending on the abnormalities considered (Table 1 and Table 2). According to Brescia et al. [11], its presence (among children with and without CHD) was associated with an increased risk of death. In the study by Czosnek et al. [33], decreased systolic function of the left ventricle was associated with an increased ventricular ectopy.

### 3.4. Coexistence with Other Heart Diseases

Children with LVNC and coexisting congenital heart diseases were included in 15 studies; in six of them, it was impossible to define whether deaths and cardiac transplants occurred among children with or without CHD. In six studies, children with heart defects were excluded from the analysis (Table 1), and in two studies, it was not clearly stated whether children with CHD were included. The mortality rate among children with LVNC and CHD in comparison to children with isolated LVNC was similar (14.2% vs. 13.3%); the percentage of children who underwent cardiac transplant was 4.4% vs. 6.7%, respectively. Both studies that were only on children with isolated LVNC, as well as in those including LVNC with congenital heart diseases decreased left ventricular systolic function, as well as enlarged left ventricular end-diastolic dimension, were associated with worse outcomes.

In six studies, the coexistence of various CHDs with LVNC was analyzed, and in most of them, it was associated with adverse outcomes (Table 1 and Table 2). In the study by Ramachandran et al. [32], the coincidence of LVNC and CHD was a risk factor for longer hospitalization, as well as a higher perioperative complication rate in the pediatric population. Hirono et al. [13] outlined that children with LVNC and VSD (ventricular septal defect) tended to have lower LVEF and more often presented with congestive heart failure than patients with VSD only. Similarly, in the study by Pignatelli et al. [34], children with LVNC and Ebstein’s anomaly tended to have a worse prognosis and lower LVEF than patients with CHD only, while in the study by Hughes et al. [35], patients with LVNC and single ventricles had worst outcomes in terms of mortality. Punn et al. [19] also outlined that death or heart transplant was more common among patients with severe congenital heart diseases. In the study by Lilje et al. [36], in which children with LVNC were observed for 12 months, no statistical significant difference in terms of mortality was observed between children with coexisting CHD and children with isolated LVNC; thus, in both groups, the disease progressed as the number of patients with congestive heart failure increased during the observation period from 41% to 68%.

In three studies, the issue of the coexistence of another phenotype of cardiomyopathy as an unfavorable risk factor was raised (Table 1). According to Brescia et al. [11], children with mixed phenotypes, such as LVNC/HCM/DCM, had worse prognosis in comparison to patients with LVNC with preserved LVEF or LVNC/HCM phenotype. In the study by Shi et al. [7], children with mixed LVNC-DCM phenotype had a 2-fold higher risk of death than patients presenting with only DCM. In a study on 348 patients, Howard et al. [24] also outlined dilated phenotype as a risk factor for cardiac dysfunction in a univariate analysis. The coexistence of another phenotype of cardiomyopathy was outlined as a risk factor for unfavorable outcome only among patients without coexisting congenital heart diseases.

### 3.5. Other Risk Factors

In the study by Wang et al. [10] on 205 children with LVNC without CHD, it was shown that children with congestive heart failure at diagnosis are at greater risk for death, a heart transplant, or ICD implantation. Hirono et al. [13], in their study on children with LVNC and CHD, concluded that the presence of heart failure, not necessarily at diagnosis, is a risk factor for death in this group. However, in studies by Gan et al. [21], Shi et al. [7], and Hirono et al. [12] (on school children), patients symptomatic at diagnosis were not at greater risk.

Furthermore, younger age at diagnosis, defined by different authors variously (<1 year old, <84 months, or comparison between median age between benign and adverse groups), was associated with unfavorable outcomes in studies by Hirono et al. [12], Arunamata et al. [15], van Waning et al. [16], Wang et al. [10], Brescia et al. [11], and Tsai et al. [22] (studies on both children with and without congenital heart diseases as listed in Table 1 and Table 2). However, in reports by Shi et al. [7] on children with isolated LVNC and Punn et al. [19] (including children with CHD) age at diagnosis was not associated with adverse outcomes.

## 4. Discussion

### 4.1. The Role of Echocardiography

Echocardiography is a basic tool used in the diagnosis of LVNC. Multiple diagnostic criteria have been outlined, with those according to Jenni et al. [25] (presence of multiple trabeculations, deep intertrabecular recesses, and thickness of the noncompacted to compacted myocardium in systole (NC/C) > 2:1) are the most commonly used in the pediatric and adult populations; thus, it is of note that in the original criteria congenital heart diseases excluded the diagnosis of LVNC [25,37]. Some authors suggest that in children, the criteria according to Chin et al. [30] (ratio between the epicardial surface to trabeculation base and epicardial surface to trabeculation peak in end-diastole [X/Y]) are more reliable [38]. Other diagnostic criteria that were found in the reviewed literature included those according to Stollberger et al. [31] (presence of >3 trabeculations at end-diastole, which moved synchronously with the compacted myocardium and presence of perfusion on color Doppler of the intertrabecular spaces) and Ichida et al. (NC/C > 2 measured at end-diastole, deep intertrabecular recesses with blood flow visualized on color Doppler)—the latter were mentioned in publications by Hirono et al. [12,13]. However, the original article was impossible to trace. Altogether four different diagnostic echocardiographic criteria and their combinations were used in the studies included in this review, which underlines the indisputable need for international, consistent guidelines concerning the diagnosis of LVNC in pediatric patients. Even though the criteria overlap with each other, some require measurement of the intertrabecular recesses in systole, while others in diastole. Moreover, there has been no consensus regarding the echocardiographic projections; some measure the NC/C ratio in the short axis, while others in the apical view, which further underlines the need for international consensus.

Adult patients with LVNC and lower left ventricular ejection fraction (LVEF) are at higher risk of adverse cardiovascular events [39]. Likewise, in the pediatric population cardiac dysfunction is a risk factor for ventricular arrhythmia, death, or transplantation [7,11,13,17,28,33]. In a retrospective study by Brescia et al. [11], out of 242 children diagnosed with LVNC, 62% presented with or developed systolic ventricular dysfunction, which delineates the severity of the problem.

Unlike in the adult group, in which no correlation between the severity of trabeculations and death was found, the data concerning the role of NC/C ratio are inconsistent in the pediatric population and require further research [39]. LVPWC (left ventricular posterior wall compaction) seems to be another interesting echocardiographic parameter that warrants exploration because decreased posterior wall compaction was correlated with an increased cardiovascular risk and decreased ejection fraction of the left ventricle [10]. In one study by Hirono et al. [13] on children with LVNC and ventricular septal defects, a significant decrease of LVPWC was observed over time; however, the significance of this finding is yet to be determined.

Speckle tracking imaging in adults may be beneficial in differentiating patients with LVNC and normal EF from healthy individuals with LVNC-like traits because in a study by Cortes et al. [40], rigid body rotation (RBR) (rotation at the basal and apical level in the same direction instead of typical clockwise rotation at the basal and anti-clockwise at the apical level) was observed in some patients with LVNC with EF>50%, and is correlated with decreased global longitudinal strains (GLS). This sheds new light on possible future research directions, especially considering that children with LVNC and adverse outcomes had significantly lower values of radial, circumferential, and longitudinal strains [15].

Echocardiography, with its wide accessibility, seems to be a valuable tool in delineating children at higher risk. Regardless of decreased LVEF, lower LVPWC, higher LVEDD, and lower myocardial strain as risk factors for adverse outcomes in children, it is worth mentioning that thromboembolic events are indisputably associated with decreased EF. The importance of prophylaxis implementation is of note because this complication may also occur among pediatric patients [10].

### 4.2. The Role of CMR

The most common CMR criteria used for diagnosis of LVNC both in the adult and pediatric populations are the one by Petersen et al. (NC/C ratio > 2.3 in end-diastole) [27]. The role of CMR has been expanding, especially in light of reports concerning low reproducibility of echocardiography in LVNC diagnosis, as well as the superiority of CMR over echocardiography in detecting LVNC and assessing its spatial morphology and the extent of trabeculations [41,42,43,44]. On the other hand, the current criteria are imperfect because they may lead to overdiagnosis. In the study by Weir-McCall et al. [45], up to 20% of 1651 adult participants fulfilled one criterion for LVNC. Conversely, when extended to four diagnostic criteria (long axis compaction at end-diastole ≥2.3, short axis compaction in diastole ≥3, and in systole ≥2, noncompaction myocardial mass >20%) less than <1% were diagnosed with LVNC [45]. This underlines the importance of performing additional exams in patients with borderline criteria.

The data concerning the accuracy of echocardiography and CMR in the pediatric population are limited and inconsistent; in some studies, LVNC was diagnosed more commonly in echocardiography, in others, the superiority of CMR was outlined [46,47]. Undisputedly, one should consider the complementary value of both methods in diagnosing LVNC in the pediatric population, especially when borderline cases are at stake. Possibly innovative developing techniques in echocardiography, such as 3-dimensional echocardiography and speckle-tracking, combined with CMR, may be of use in determining the diagnosis and differentiating normal variants from pathological changes.

The segmental involvement in the pediatric population is similar to the one observed in adults; unsurprisingly, wider involvement is associated with lower ejection fraction [48]. In the adult population, patients with late gadolinium enhancement (LGE) presented with worse outcomes than those without [49]. In a meta-analysis by Grigoratos et al. [49], no unfavorable outcomes were observed in adult patients with LVNC, normal LVEF, and negative LGE. In contrast to the adult population, the role of LGE as a predictor of arrhythmia risk in the pediatric population is questionable. The different results obtained by Cortez et al. [26] and Cheng et al. [29] may be explained by the different ages of patients analyzed, with adolescents presenting more frequently with LGE. This seems to be in agreement with reports that LGE does not occur in neonates and young children with LVNC; thus, LGE may be a valuable tool in risk stratification in older patients, whereas the search for other traits in the younger population needs to be continued [50].

### 4.3. ECG

Electrocardiogram (ECG) is an easily accessible tool that is valuable not only in the diagnosis, as was indicated in a Japanese study where 42% of pediatric patients with LVNC were diagnosed, due to school ECG screening, but also in outlining higher risk patients with LVNC [11,12]. Some differences in ECG between adult and pediatric populations can be outlined; the most common ECG abnormalities in the adult population despite ventricle hypertrophies and depolarization abnormalities were bundle branch blocks and AV blocks, which were not as common in the pediatric population [37]. In adults, QTc prolongation in ECG has been observed in up to 44% of patients and has been associated with lower LVEF, increased fibrosis, as well as unfavorable prognosis [51]. The frequency of prolonged QTc interval in ECG in children varies between 9–40% (in our review 9–11%); however, its meaning has not been determined [10,11,22,52]. The differences in ECG abnormalities in the two populations suggest that adult data cannot be routinely applied to the pediatric population; furthermore, ECG aberrations possibly change with age and progression of LVNC or may be associated with the distinct underlying pathophysiology of the disease, because genetic mutations are more common in children than in adults [16].

It is of note that the occurrence of malignant arrhythmia (such as sustained ventricular tachycardia (sVT) or ventricular fibrillation (VF)) may be an independent risk factor for unfavorable outcomes, because adult patients with LVNC after ICD implantation as a secondary prophylaxis commonly presented with normal ejection fraction [53]. A similar trend was observed in children. In the study by Brescia et al. [11], not all patients who experienced sudden cardiac death had ventricular dysfunction. These findings point toward a subgroup of patients with LVNC, who have preserved cardiac function and concomitant malignant arrhythmias, and for this reason, require special attention. Genetic testing might be of use in outlining higher risk patients, because mutations in genes that are typically associated with channelopathies, such as long QT-interval or catecholaminergic VT, have been detected in patients with LVNC; interestingly, in some patients, features of LVNC were not present at diagnosis and developed with time [6].

### 4.4. Coexistence with Other Heart Diseases

Because LVNC is heterogeneous in its image, it may coexist with other types of cardiomyopathy or progress to a mixed form, which was observed in 12% of cases within two years in the study by Jefferies et al. [2] on the cardiomyopathy registry in the USA and Canada. Some authors suggest that the nomenclature should include prior cardiomyopathy with coexisting LVNC traits because the latter describes only morphological features; this could simplify the classification and help to delineate higher risk patients [54]. Nevertheless, children with LVNC concomitant with other phenotypes of cardiomyopathy frequently present with heart failure and have a worse prognosis, among whom children with coexisting DCM or indeterminate cardiomyopathy present with the worst outcomes [2]. This seems to be in agreement with the observations made by some authors that greater LVEDD was associated with worse outcomes in children [7,13,15,17,23]. The burden of the need for clear classification of LVNC prevails, as some authors outlined mixed forms of LVNC, such as LVNC-DCM type, while others pointed out only echocardiographic traits characteristic for DCM, i.e., increased LVEDD. It is of note that, in contrast to the pediatric population, worse outcome of patients with mixed phenotype has not been confirmed unequivocally in adult studies, which once again points toward possible differences in adult and pediatric LVNC [9,55].

Furthermore, LVNC can coexist with different congenital heart diseases (CHD), varying from mild types, such as patent ductus arteriosus or septal defects to more severe, i.e., Ebstein’s anomaly or hypoplastic left heart syndrome (HLHS) [5]. Familial occurrence has been reported with some family members presenting with pure LVNC, while others with concomitant CHD [6]. Patients with CHD and LVNC tend to have a worse prognosis, require longer hospitalizations, and more frequently present with postoperative complications than children with CHD only, which points toward a sub-group of patients requiring more attention [13,32]. However, a large meta-analysis concerning the survival of children with isolated LVNC, as well as those with coexisting CHD, is necessary to draw clear conclusions, as comparing mortality and transplantation rates from 16 studies showed no significant differences between the two groups. It is of note that LVNC has been reported to occur more frequently among children with heterotaxy syndrome, with a prevalence of 7.5% vs. 0.013–1.3% in the general population, which may suggest a common genetic mechanism [56].

### 4.5. Other Risk Factors

#### Influence of Medical Treatment

Reports concerning the effect of medical treatment on ECG and echocardiography are inconsistent. In a small study on 20 adults, 13-month treatment with β-blockers did not significantly influence the ECG or the LVEF; however, it led to a reduction in the LV mass [57]. In scarce pediatric reports, medical therapy with angiotensin-converting enzyme inhibitors (ACE-I), angiotensin II receptor blockers, β-blockers, or combinations of the former has been associated with an improvement of the ejection fraction and a decrease in the size of the left ventricular diastolic dimension, which points towards a favorable remodeling effect [58,59]. Treatment with carvedilol has been shown to improve left ventricular function; however, the long-term influence of medication on the survival of children with LVNC has not been assessed [23].

Due to familial occurrence of LVNC in around 30% and familiar history of SCD in 18% of children with LVNC, genetic testing, and detailed familiar background may help determine higher risk patients with accidentally found LVNC [2,10,60]. A positive genetic profile is more common in children than in adults, with the latter more likely to have sporadic LVNC, whereas in children, abnormalities in chromosomes, and x-linked and mitochondrial genes are more prevalent [9,16]. Moreover, in contrast to adults, genetically confirmed pediatric LVNC has been associated with worse outcomes [16].

Among family members of patients with different cardiomyopathies hypertrabeculation of the myocardium is the most common abnormality, which suggests that noncompaction is a morphological finding that can develop into cardiomyopathy in the future, and that the formation of LVNC is a continuous process [61]. Conversely, due to the presence of healthy subjects with trabeculations in the LV, genetic profiling may assist in identifying pathogenic mutations and in differentiating subjects at risk. Knowledge of the genetic background may be of importance in determining the progression of the disease because certain mutations in genes, i.e., DMD (Duchene muscular dystrophy), are associated with the dynamic course of the disease and progression in the trabeculations and severity of heart failure time [6]. Waning et al. [9], in their systematic review on adults and children, outlined that the presence of mutations in some genes (such as MYBPC3, TTN, arrhythmia, and nonsarcomere nonarrythmia genes and X-linked genes) was associated with an increased risk of adverse events, whereas patients with most common mutations in MYH7 were at a lower risk. Furthermore, genetic testing may help outline patients at greater risk of severe heart failure because the presence of genetic mutations in genes associated with cardiomyopathies has been linked to lower ejection fraction [16,62]. In a study on adults and children, a greater number of genetic variants of interest (VOI) was associated with lower LVEF and greater NC/C ratio in MRI [63].

Unequivocally, the current criteria for the diagnosis of LVNC require revision. Perhaps it would be reasonable to include abnormal ventricle function, the presence of arrhythmia, as well as genetic information into the scheme in the future. Conversely, one must keep in mind the risk of overdiagnosis, especially in the adult population [45]. The AHA/ACC recommendations for competitive athletes with cardiovascular diseases do not restrain asymptomatic patients with LVNC with normal left ventricular systolic function, without significant ventricular arrhythmias, and without unexplained syncope from participation in competitive sports [64]. Similar conclusions have been drawn in the pediatric population, because patients with such characteristics are perceived to have a low risk of sudden cardiac death, and for this reason, they are not restricted from sporting activities [11].

### 4.6. Study Limitations

The limitations of the study include selection bias and possible duplication of data because some reports were from the same centers, but from different years. Furthermore, due to the lack of international consensus, we included studies that used different diagnostic criteria for LVNC, which may result in an increased diversity of the groups presented. Another limitation is the lack of statistical metanalysis; heterogeneous data, as well as different cut-off values and approaches to data presentation (i.e., different echocardiographic parameters measured, with LVEF presentation ranging from LVEF<55% to LVEF<24%, or some authors calculating LVEF as a continuous variable) restricted the statistical analysis.

## 5. Conclusions

ECG and imaging tests, such as echocardiography and magnetic resonance, help outline risk factors for unfavorable outcomes of LVNC in children, as well as dividing patients into subgroups at risk, such as the following: Those with known genetic mutations, coexisting cardiomyopathies, congenital heart diseases, decreased EF, greater LVEDD, as well as patients with ECG abnormalities and arrhythmia. It is noteworthy that some differences between adult and pediatric LVNC in terms of ECG, echocardiographic, CMR, and genetic test results can be outlined, with the latter presenting with worse outcomes when mixed cardiomyopathy traits or genetic mutations are present. Increased genetic testing will help to improve the knowledge concerning genetic variants and assist in identifying more patients at risk.

Undoubtedly, the burden of pediatric LVNC prevails, and an international consensus is essential, because the current diagnostic criteria are inconsistent and do not unequivocally point outpatients who do not require special attention from health care professionals and can safely undertake physical activity.

## Figures and Tables

**Table 1:** Characteristics of patients without congenital heart diseases, as well as echocardiographic, CMR, and other risk factors for unfavorable outcomes.

		Diagnostic Criteria	N	Age Median in Years (IQR)	Deaths/ HTx	Arrhythmia n	Echocardiographic Findings and Risk Factors	CMR Findings and Risk Factors	Other Risk Factors
1	Howard et al., 2019 [24]	Jenni [25]	348	6.8 (0.5–13.8)	31/ 20	SVT—27 (17 in patients with WPW)	lower LVEF in patients with LVNC and WPW than without WPW	-	- No difference in terms of survival between patients with and without WPW - WPW risk factor for cardiac dysfunction
2	Cortez et al., 2019 [26]	Petersen [27]	39	1 (0.8–3) with VA 0.5 (0.2–12) without VA	-	VA—8	-	- LVEDVi greater in patients with VA - LGE was not a predictor of VA	-
3	Shi et al., 2018 [7] /Bharucha et al., 2015 [28]	Jenni [25]	29	age at diagnosis 0.3 (0.1–1.3)	14 /6	-	* lower FS z-score * larger LVEDD z-score greater LV NC/C in diastole (*p =* 0.07)	-	* LVNC-D worse prognosis than DCM * sporadic LVNC (nonfamilial) * female sex
4	Wang et al., 2017 [10]	Ichida [12]	205	2.7 mo infantile group 7.3 y in juvenile group	23 /9	total—20 VT—11 SSS—5 AF/AFl—4 SVT—5	* lower LVPWC z-score ***(≤−1.5)*** * LVEF<50% in juvenile	-	* CHF at diagnosis * age at onset
5	Cheng et al., 2015 [29]	Petersen [27]	40	mean 13.7 ± 3	6/ 2	VT/VF—7	-	* LGE+	-
6	Brescia et al., 2013 [11]/ Jefferies et al., 2015 [2]	Jenni [25]	242	9 (3 mo–13.8)	31 /13	* arrhythmia total—81 VT/VF—42 atrial tachycardia—14 SVT—19 Afl—4	* LVEF < 55%	-	* age at presentation < 1 y * LVNC/HCM/DCM phenotype worse prognosis than LVNC with preserved EF, or LVNC/HCM

* Risk factors for unfavorable outcomes of LVNC have been bolded and highlighted with *. *Justified*—risk factors in multivariable analysis. N—number of patients; mo—months; CHF—congestive heart failure; LVEF—left ventricular ejection fraction; LVEDD—left ventricular end-diastolic dimension; FS—fractional shortening; LVPWC—left ventricular posterior wall compaction; NC/C—noncompaction/compaction ratio; LGE—late gadolinium enhancement; LVEDVi—indexed left ventricular end-diastolic volume; VT—ventricular tachycardia; VF—ventricular fibrillation; SVT—supraventricular tachycardia, Afl—atrial flutter; AF—atrial fibrillation; VA—ventricular arrhythmia; SSS—sinus sick syndrome; WPW—Wolf Parkinson White syndrome; HTx—heart transplant; DCM—dilated cardiomyopathy; HCM—hypertrophic cardiomyopathy.

**Table 2:** Characteristics of patients with congenital heart diseases, as well as echocardiographic, CMR, and other risk factors for unfavorable outcomes.

		Diagnostic Criteria	N	Age Median in Years (IQR)	Deaths/ HTx	Coexisting CHD n	Arrhythmia n	Echocardiographic Findings and Risk Factors	CMR Findings and Risk Factors	Other Risk Factors
1	Hirono et al., 2020 [13]	Ichida [12]	53	0.3 mo (range 0–14y)	4 /0	53	Total—13 VT—5 SVT—2 AFL—2	* LVEF < 24% * LVEDD z-score > 8.56 * NC/C at apex > 8.33	-	* heart failure -children with LVNC and VSD lower EF and more often CHF than children with VSD alone
2	Hirono et al., 2020 [12]	Ichida [12]	105	7.3 (range 0–16.4)	4 /1	Other cardiac defects—6	PVC—22 VT—1 other—13	* EF < 55%	-	* treatment with B-blockers * younger age at diagnosis (<84 m) * symptomatic after treatment
3	Rodriguez-Fanjul et al., 2020 [14]	Jenni [25] and Chin [30]	14	Neonates	6 (5 with CHD) /0	13	VF—1 WPW—1	* severely depressed systolic function or biventricular involvement	-	-
4	Gan et al., 2020 [21]	Stollberger [31]	124 (47 with i-LVNC)	NC/C < 2 7.2 (2.2–34 mo) NC/C > 26.8 (3.5–44.5 mo)	15 /0	77	-	* NC/C > 2 worse survival in i-LVNC no association between survival and baseline EF	-	-
5	Arunamata et al., 2019 [15]	Jenni [25], Stollberger [31] and Chin [30]	101	2.8 (range 0–19.4)	14 /16	44	-	* lower LVEF, FS * greater LVEDD and LV mass z-score * decreased global radial, circumferential and longitudinal strain	-	* younger age at diagnosis
6	van Waning et al., 2018 [16]	Jenni [25] and Petersen [27]	327 (52 children)	7 (0–14)	8 /4	14	AF—5 sVT/VF—3	* **LV systolic dysfunction** (no differentiation between echo an CMR results)		* genetic and probable genetic LVNC in children * multiple mutations in MYBPC3 * diagnosis < 1 yr
7	Ramachandran et al., 2016 [32]	Jenni [25]	26	0.24 (0.01–0.86)	3 /1	26	perioperative arrhythmias—7 CAVB—4 VT/VF—2	-	-	LVNC with CHD longer hospitalization and higher perioperative complications rate
8	Czosnek et al., 2015 [33]	n/s	72	mean 13	1 /0	n/s	nsVT—3 PVC—37 FAT—1 conduction system disease—1	-	-	Ventricular ectopy more often in patients with EF < 55%
9	Pignatelli et al., 2014 [34]	n/s	10 with Ebstein+ LVNC	Neonates	3 /0	10	-	higher risk of progressive LV dysfunction in patients with LVNC and EA than EA alone	-	* higher risk of adverse outcomes in patients with LVNC and EA than EA alone
10	Zuckerman et al., 2011 [17]	n/s	58	0.3 (range 1d–21y)	11 /15	13	-	* lower FS * greater LVEDD	-	* hemodynamic instability (requiring mechanical support/inotropic agents)
11	Ozgur et al., 2011 [18]	n/s	29	mean 4.8 ± 4.6	6 /n/s	7	total—8 PVC—5 VT—1	* lower LV systolic function at diagnosis (p-value 0.058)	-	-
12	Punn et al., 2010 [19]	Jenni [25]	44	range 1d–16y	7 /9	22	VT—2	* more segments involved * lower LVEF, FS	-	LVNC with significant CHD
13	Tsai et al., 2009 [22]	Chin [30]	46	0.4 (range birth—18.5)	9 /0	36	SVT—3 VT—2 junctional rhythm-3 ectopic atrial rhythm—2	no association between mortality and EF	-	* lower age at diagnosis
14	McMahon et al., 2007 [20]	Jenni [25] and Stollberger [31]	56	4.8 (range 0.3–18)	8 /4	7	total—13 VT—6 SVT—2 AET—3 AF—1 CAVB—1	* *reduced lateral mitral e’ velocity* * septal e’ velocity * lateral mitral E/e’ * lower LVEF	-	-
15	Hughes et al., 2007 [35]	angiography	31	(range 1 day–2 years)	3 /2	31	-	-	-	* presence of noncomapction * LVNC and single ventricle—worst outcomes
16	Lilje et al., 2006 [36]	Chin [30]	66	4 (range 0–21)	4 (1 with CHD) /n/s	41	total—4	-	-	no difference in terms of mortality between LVNC patients with and w/o CHD
17	Wald et al., 2004 [23]	Jenni [25]	22	mean 3.9 (range 0–16)	3 /2	n/s	AA—2 VA—5	* increased LVEDD at presentation * NC/C ratio > 3	-	-

* Risk factors for unfavorable outcomes of LVNC have been bolded and highlighted with *. *Justified*—risk factors in multivariable analysis. n/s-not exactly specified. N—number of patients; mo—months; CHD—congenital heart diseases; CHF—congestive heart failure; LVEF—left ventricular ejection fraction; LVEDD—left ventricular end-diastolic dimension; FS—fractional shortening; NC/C—noncompaction/compaction ratio; VT—ventricular tachycardia; sVT—sustained ventricular tachycardia; nsVT—nonsustained ventricular tachycardia; SVT—supraventricular tachycardia; CAVB-complete atrioventricular block; AFL—atrial flutter; AF—atrial fibrillation; AA—atrial arrhythmia, VA—ventricular arrhythmia; PVC—premature ventricular contraction; AET—atrial ectopic tachycardia; WPW—Wolf Parkinson White syndrome; FAT—focal atrial tachycardia; i-LVNC—isolated LVNC; HTx—heart transplant; VSD—ventricular septal defect; EA—Ebstein anomaly.

**Table 3:** Most common ECG abnormalities and risk factors for unfavorable outcomes.

	N	Abnormal ECG	Abnormal T-Wave	Abnormal ST	Fragm. QRS	J Wave	VH	RBBB	LBBB	WPW/ Preexciation	LQT	Other	Risk Factors
Hirono et al. [13]	53		8	3	16	7		10	3	1	5	pathologic Q wave—7 AVB 3rd—4	
Hirono et al. [12]	146		6	2	49	23		16	4	0		axis deviation—18 pathologic Q wave—1 AVB—1	* T-wave abnormality in first graders
Rodriquez-Fanjul et al. [14]	14	9		2			6			1			
Gan et al. [21]	47	27 ^1^											
Howard et al. [24]	348									38			
Cortez et al. [26]	38		33/23 ^2^									AVB 3rd—1	* spatial peak QRS-T angle > 147 degrees
van Waning et al. [16]	52							5	1			AVB 1st—2 brady—3	
Wang et al. [10]	205	115							7	10	3	AVB 3rd—8	
Cheng et al. [29]	40	36											
Brescia et al. [11]	242	210	94	82			100		1	20	22	atrial enlargement—46 left axis deviation—22	* ST abnormality * T-wave inversion
Tsai et al. [22]	35	28 ¹					19/15 ^3^	2		3	4	AVB 1st—4 interventricular conduction delay-3	
Wald et al. [23]	22	22	3						1	1		enlarged chamber dimension—16	

* Risk factors for unfavorable outcomes of LVNC have been bolded and highlighted with *. VH—ventricular hypertrophy; RBBB—right bundle branch block; LBBB—left bundle branch block; WPW—Wolf Parkinson White syndrome; LQT—long QT; AVB atrioventricular block, brady—bradycardia; ^1^ abnormal ECG and arrhythmia together. ^2^ division into later and inferior abnormalities of the T-wave. ^3^ Right VH/Left VH.

## Data Availability

Data is contained within the article.
